# The *aphA1* kanamycin and neomycin resistance gene originated in *Klebsiella michiganensis*

**DOI:** 10.1093/jac/dkaf372

**Published:** 2025-10-03

**Authors:** Robert A Moran, Ruth M Hall

**Affiliations:** School of Life and Environmental Sciences, The University of Sydney, Sydney, New South Wales, Australia; School of Life and Environmental Sciences, The University of Sydney, Sydney, New South Wales, Australia

## Abstract

**Background:**

The origins of several antibiotic resistance genes have been traced to intrinsic genes present in bacterial chromosomes. The *aphA1* gene, which confers resistance to the aminoglycosides kanamycin and neomycin, is commonly found in diverse Gram-negative bacterial pathogens, and is associated with several different mobile genetic elements. However, its origin had not been identified.

**Objectives:**

To determine whether the *aphA1-*containing segments in novel compound and pseudo-compound transposons found in three historic plasmids, pIE545, R478 and Rts1, are found in the chromosome of a specific bacterial species.

**Methods:**

Passenger segments of transposons containing the *aphA1* gene were compared to one another and to chromosomal sequences in GenBank using BLAST.

**Results:**

In pIE545, *aphA1* is in a 5424 bp compound transposon, Tn*aphA1*-pIE545, that is bounded by directly oriented copies of IS*102*. The 3.3 kb *aphA1*-containing passenger segment is >99% identical to a contiguous part of several *Klebsiella michiganensis* chromosomes. The 4.3 kb *aphA1*-containing segment of the IS*26*-bounded pseudo-compound transposon PTn*aphA1*-R478 in R478 is also >99% identical to a contiguous part of several *K. michiganensis* chromosomes that differ in the gene content surrounding *aphA1*. The 2.2 kb passenger segment of the IS*26*-bounded PTn*2680* from Rts1 arose via a third independent acquisition from a *K. michiganensis* chromosome.

**Conclusions:**

The *aphA1* gene has been captured and mobilized on at least three occasions from *K. michiganensis* chromosomes with related but distinct surrounding configurations.

## Introduction

The source of a number of antibiotic resistance genes found in Gram-negative bacterial pathogens have been identified. For example, it has been known for over 20 years that the *bla*_SHV_ gene is derived from the *Klebsiella pneumoniae* chromosome,^1^ having been picked up by IS*26* in two separate events to form the now widespread pseudo-compound transposons (PCTs) known as TnSHV-long and TnSHV-short.^2^ Similarly, *bla*_CTX-M_ genes are derived from a *Kluyvera* chromosome, and other resistance genes have been traced to origins in various members of the Enterobacterales.^3–5^ Most of these resistance genes have been captured and mobilized by IS to form composite/compound or PCTs (two IS copies flanking a central passenger segment) or transposition units (one IS and an adjacent passenger segment in the case of ISEcp1).

The *aphA1* gene that confers resistance to the aminoglycosides kanamycin and neomycin has been found in plasmids in some of the earliest antibiotic-resistant isolates investigated.^6^ It is present in the IncC plasmid pIP40a from a *Pseudomonas aeruginosa* isolated in France in 1969,^7^ in the IncB/O plasmid R805a from a *Salmonella enterica* Typhi isolated in Mexico in 1972,^8^ and in the IncT plasmid Rts1 from a *Proteus vulgaris* isolated in Japan and first reported in 1967.^9^ The *aphA1* gene is also widespread in modern multi-drug-resistant Gram-negative bacterial pathogens, even though neither kanamycin nor neomycin are currently used therapeutically. For example, many plasmids of the broad host range A/C and L/M complexes carry *aphA1*,^10,11^ and both GC1 and GC2 *A. baumannii* isolates often carry *aphA1* in their chromosomal resistance islands.^12–14^ The *aphA1* gene has been found associated with a number of IS, such as IS*903*,^15^ IS*602*^16^ and IS*26*.^17^ However, a potential source for *aphA1* has never been found.

Here, we have identified a new *aphA1*-containing composite transposon in the historic IncZ plasmid pIE545 that was recovered from a 1970s *K*. *pneumoniae* isolate.^18^ We examined the passenger segments of this and two other *aphA1*-carrying transposons and found that they are each almost identical to a region in a different group of *Klebsiella michiganensis* chromosomes.

## Materials and methods

### Sequence analysis

Plasmid pIE545 was recovered from *Escherichia coli* JP1290 that had been stored freeze-dried in the collection of Professor Ron Skurray at the University of Sydney since 1990. Plasmid DNA was isolated and sequenced on the Illumina platform and reads were assembled using SPAdes v3.5.0.^19^ The sequence was closed with gap-spanning PCRs, and assembly of the contigs and Sanger-sequenced PCR products was performed in Sequencher (v4.10; Gene Codes Corporation). The complete sequence of pIE545 is available under GenBank accession PV671756 and will be described elsewhere. Insertion sequences were identified using ISFinder.^20^ Sequences of passenger segments from transposons carrying *aphA1* were compared to one another and queried against the GenBank non-redundant nucleotide database using BLAST.^21^

### Nomenclature

In order to retain the connection to the extensive historic and current literature on the *aphA1* gene, we have used the original name *aphA1* throughout this manuscript, rather than the awkward and poorly understood *aph(3′)-Ia* (or *-Ib*) currently used by the various resistance gene databases.

## Results and discussion

**Figure 1.**
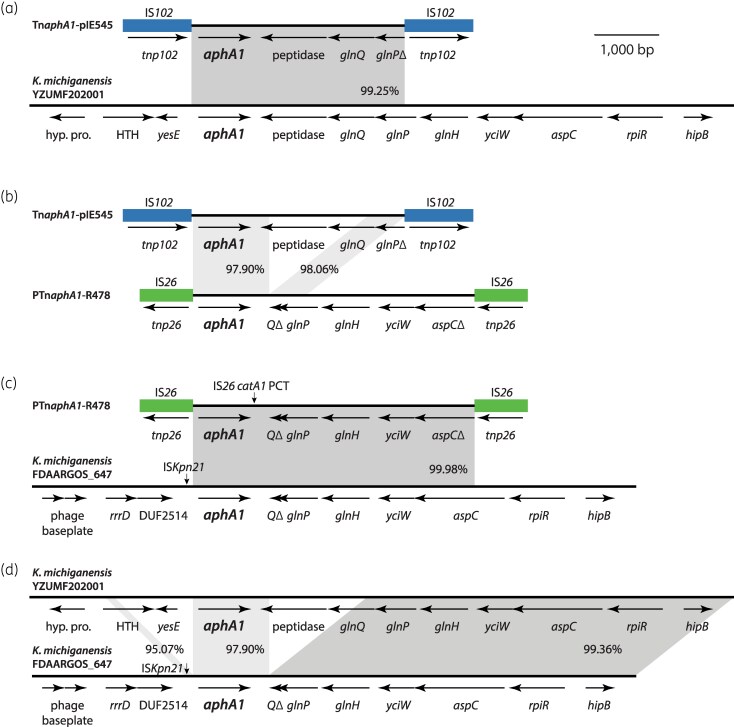
Contexts of the *aphA1* gene. Schematic diagrams showing the conservation of *aphA1*-containing passenger segments relative to one another and to *K. michiganensis* chromosomes. The extents and orientations of open reading frames are shown as labelled arrows below the lines that represent DNA sequences. Insertion sequences IS*102* and IS*26* are shown as labelled boxes. The shading between DNA sequences represents conservation, with nucleotide identities (%) shown for each block. (a) Tn*aphA1*-pIE545 and *K. michiganensis* YZUMF202001. (b) Tn*aphA1*-pIE545 and PTn*aphA1*-R478. (c) PTn*aphA1*-R478 and *K. michiganensis* FDAARGOS_647. (d) *K. michiganensis* YZUMF202001 and FDAARGOS_647. Drawn to scale from GenBank accessions PV671756, BX664015, CP097554 and CP044109.

The *aphA1* gene in pIE545 was found in a 3312 bp segment located between directly oriented copies of the IS*5* family element IS*102* (Figure 1a). This 5424 bp transposon, Tn*aphA1*-pIE545, is flanked by a 9 bp target site duplication (TSD). The *aphA1* start codon is 116 bp away from the end of the upstream IS*102* and a potential promoter (TTGTGT-[17]-TACATT) was found in this span. The passenger segment also includes a gene for a putative C45 family peptidase, the glutamine ABC transporter gene *glnQ*, and a partial *glnP* that has been truncated by IS*102*.

Using the 3312 bp central segment to query the GenBank non-redundant nucleotide database returned contiguous matches to several entries for *K. michiganensis* chromosomes. The most closely related was that of strain YZUMF202001 (accession CP097554), which was isolated from a corn smut tumour in China in 2020,^22^ and is 99.25% identical (3297/3322 bp) (Figure 1a). In YZUMF202001, the *glnP* is complete and together with *glnQ* and the upstream *glnH* encodes a putative glutamine ABC transporter. No IS were detected in the 10 kb upstream and downstream of the *aphA1* gene in YZUMF2020001. Given that the sequences in Tn*aphA1*-pIE545 and YZUMF202001 differ by only 15 single nucleotide differences and a 10 bp gap we conclude that a *K. michiganensis* strain was the source of the Tn*aphA1*-pIE545 passenger segment.

Previously, we found the longest IS*26*-associated *aphA1*-containing passenger segment in the IncHI2 plasmid R478.^23,24^ R478 is also a historic plasmid, from a *Serratia marcescens* isolated in the USA in 1969.^25^ The *aphA1* in R478 is part of an IS*26*-bounded PCT, here named PTn*aphA1*-R478, that in R478 is interrupted by another IS*26*-bounded PCT that contains the chloramphenicol resistance gene *catA1*.^23^ With the 5388 bp *catA1* PCT and one copy of the 8 bp TSD removed, the *aphA1*-containing passenger segment is 4364 bp (Figure 1b). This segment includes a partial *glnQ*, *glnPH*, *yciW* and a partial *aspC* that has been truncated by IS*26*. Comparison of the *aphA1*-containing segments of Tn*aphA1*-pIE545 and PTn*aphA1*-R478 revealed that they diverge somewhat in gene content, and where they overlap share only approximately 98% nucleotide identity (Figure 1b). A 1540 bp fragment downstream of *aphA1* in Tn*aphA1*-pIE545, which includes a peptidase gene and most of *glnQ*, is not present in PTn*aphA1*-R478. Either side of the missing 1540 bp, the Tn*aphA1*-pIE545 and PTn*aphA1*-R478 passenger segments share 1215 and 557 bp regions that have nucleotide identities of 97.90% and 98.06%, respectively (Figure 1b).

When the 4364 bp central segment of PTn*aphA1*-R478 was used to query GenBank, contiguous matches to a different set of *K. michiganensis* chromosome entries were returned. The most closely related was FDAARGOS_647, which was isolated from a clinical specimen in the USA and was 99.98% identical (4363/4364 bp) (Figure 1c). Only a single copy of ISKpn21 was found in the 10 kb upstream and downstream of *aphA1* in FDAARGOS_647. While the segment containing the peptidase gene is present in YZUMF202001 and several related *K. michiganensis* chromosomes, it has been lost from FDAARGOS_647 and several other *K. michiganensis* chromosomes that are syntenic with the PTn*aphA1*-R478 segment (Figure 1d). As the deletion event that removed 1540 bp downstream of *aphA1* clearly occurred in the *K. michiganensis* chromosome prior to its capture by IS*26*, we conclude that the passenger segment of PTn*aphA1*-R478 was picked up in a second, separate event.

Several compound transposons and PCTs that include smaller central segments, usually containing only the *aphA1* gene and a small amount of surrounding sequence, have been identified and named (Table 1). After Tn*aphA1*-pIE545 and PTn*aphA1*-R478, the longest passenger segment is 2296 bp, found in the IS*26*-bounded PTn*2680*, which is interrupted by a copy of IS*903* in plasmid Rts1.^9^ With IS*903* and one copy of the TSD removed, the central segment of PTn*2680* includes 1305 bp upstream of *aphA1* that is 99.39% identical (1297/1305 bp) to a contiguous segment in a single GenBank entry, the chromosome of *K. michiganensis* Kfme267 (CP071393), which was isolated from a clinical specimen in China in 2019. However, a deletion event extending from within *aphA1* to within *rpiR* has truncated both genes in Kfme267 (Figure S1, available as Supplementary data at JAC Online). Thus, the passenger segment of PTn*2680* is likely derived from a related chromosome that is not yet represented in GenBank. As the region upstream of *aphA1* in PTn*2680* and Kfme267 differs from those in YZUMF202001 and FDAARGOS_267, it appears to represent a third *aphA1* pickup.

**Table 1.** *aphA1*-containing transposons and pseudo-compound transposons

Element	IS	Passenger segment length (bp)^a^	GenBank		References
			Accession	Co-ordinates	
PTn*aphA1*-R478	IS*26*	4364^b^	BX664015	93 341–104 740	Cain and Hall^23^; Gilmour *et al*.^25^
PTn*2680*	IS*26*	2296^c^	AP004237	114 687–119 688	Mollet *et al*.^9^
PTn*6020*	IS*26*	1254	JF343535	1–2754	Nigro *et al*.^13^
PTn*4352*	IS*26*	1040	M20306	27–2706	Wrighton and Strike^17^
PTn*6179*	IS*26*	931	KX011025	49 947–52 652	Blackwell *et al*.^14^
Tn*aphA1*-pIE545	IS*102*^d^	3312	PV671756	20 346–25 769	This study
Tn9*03*	IS*903*^d^	980	V00359	1–3094	Oka *et al*.^15^
Tn*602*	IS*602*^d^	950	MK088173	5704–8767	Moran *et al*.^8^; Stibitz and Davies^16^

^a^The *aphA1* gene is 816 bp.

^b^Excludes 5388 bp *catA1* PCT and one copy of its 8 bp target site duplication.

^c^Excludes 1057 bp IS*903* and one copy of its 9 bp target site duplication.

^d^IS*102*, IS*602* and IS*903* are closely related (>94% ID) members of the IS*903* group in the IS*5* family.

The shorter segments in the remaining transposons (931–1254 bp; see Table 1) include little more than the 816 bp *aphA1* gene and may be derived from the longer passenger segments described here. Indeed, the 1254 bp passenger segment of PTn*6020* is identical to the corresponding part of PTn*aphA1*-R478, and the 931 bp passenger segment of PTn*6179* differs from it at just one position. Other passenger segments could represent further pickup events from *K. michiganensis*.

Of the four IS that have been found associated with *aphA1*, three (IS*903*; IS*602*; IS*102*) belong to the IS*903* group in the IS*5* family (Table 1). While IS*26* has been extensively characterized over the past decade and the pathway used to pick up DNA segments and create PCTs is understood,^4^ less is known about the capacity of IS*903* group elements for gene capture and mobilization. IS*26* mediates adjacent deletions that generate circular translocatable units that can insert at new sites forming PCTs in the process.^4^ IS*903* has also been shown to generate adjacent deletions and to form cointegrates,^26^ but further experimental characterization of this element is required to clarify how it and other IS in the IS*903* group form transposons.

Identification of the source of antibiotic resistance genes depends on the availability of genome sequences for the appropriate bacterial species. Indeed, the source of the *bla*_SHV_ gene was identified shortly after the first *K. pneumoniae* genome became available over 20 years ago.^1^ The ability to identify the source of *aphA1* as described here was contingent on the more recent availability of *K. michiganensis* genomes. Similarly, *Atlantibacter hermannii* has recently been identified as the source of the chloramphenicol resistance gene *catA1*.^27^

## Conclusions

The *aphA1* gene has been mobilized from distinct *K. michiganensis* backgrounds by different IS on at least three independent occasions.

## Supplementary Material

dkaf372_Supplementary_Data

## Supplementary data

Figure S1 is available as Supplementary data at *JAC* Online.
