# Supplementary material for: The aphA1 kanamycin and neomycin resistance gene originated in Klebsiella michiganensis
Source: J Antimicrob Chemother. 2025 Oct 3;80(12):3340–4. doi: 10.1093/jac/dkaf372 (PMC12670165; doi:10.1093/jac/dkaf372)
Supplement: dkaf372_Supplementary_Data [file dkaf372_supplementary_data.pdf]

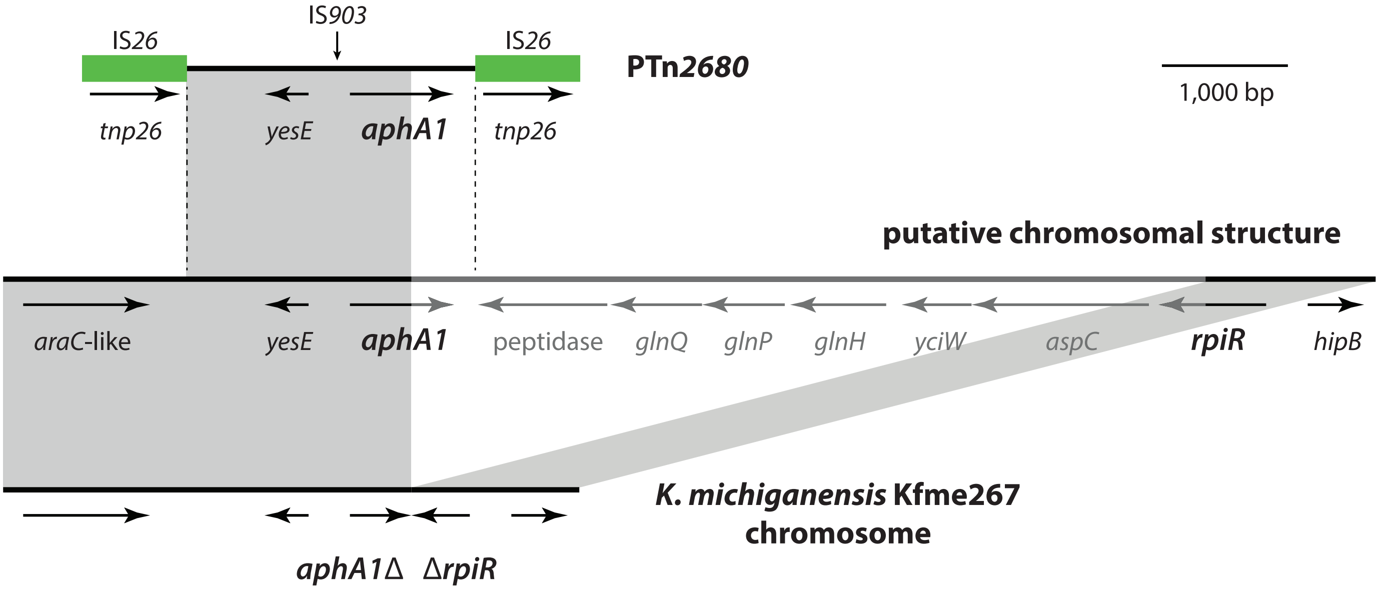

Figure S1: Putative provenance of the *aphA1* gene in PTn*2680*. Schematic showing PTn*2680* of plasmid Rts1, the corresponding chromosomal segment of *K. michiganensis* Kfme267, and a putative chromosomal structure from which the passenger segment in PTn*2680* is likely derived. The extents and orientations of open reading frames are shown as labelled arrows below the lines that represent DNA sequences. IS*26* is shown as a labelled green box. Grey shading between DNA sequences represents conservation, with nucleotide identities listed in-text. PTn*2680* and Kfme267 sequences drawn to scale from GenBank accessions AP004237 and CP071393, respectively. IS*903* and one copy of its 9 bp target site duplication were removed from the PTn*2680* passenger segment, with the IS*903* insertion site indicated by a labelled arrow. The putative chromosomal sequence was generated by adding sequence from *K. michiganensis* YZUMF202001 (GenBank accession CP097554) between the truncated *aphA1* and *rpiR* of Kfme267 to restore the structure expected to have been present before a deletion event yielded the Kfme267 structure.
